# On the generalized Hartman effect presumption in semiconductors and photonic structures

**DOI:** 10.1186/1556-276X-8-145

**Published:** 2013-03-28

**Authors:** Herbert P Simanjuntak, Pedro Pereyra

**Affiliations:** 1 Departemen Fisika, FMIPA, Universitas Indonesia, Depok, West Java, 16424, Indonesia; 2 Departamento de Ciencias Básicas, UAM-Azcapotzalco, Mexico, Federal District, C.P. 02200, Mexico; 3 Institute für Experimental and Applied Physics, University of Regensburg, Regensburg, Bavaria, Germany

**Keywords:** Hartman effect, Tunneling time, Bragg gratings, Superlattice, 03.65.Xp, 52.25.Bs, 42.70.Qs, 78.67.-m

## Abstract

We analyze different examples to show that the so-called generalized Hartman effect is an erroneous presumption. The results obtained for electron tunneling and transmission of electromagnetic waves through superlattices and Bragg gratings show clearly the resonant character of the phase time behavior so that a generalized Hartman effect is not expected to occur. A reinterpretation of the experimental results in double Bragg gratings is proposed.

## Background

The Hartman [1] effect is known as the independence of the tunneling time on the barrier width as this parameter gets large. It has been shown that the experimental evidences of this effect on the transmission times of photons and electromagnetic pulses [2-6] are compatible with phase time calculations [7]. The Hartman effect has been investigated in various ways by extending the system not only for a single barrier but also for double [8,9] and multiple barrier [10,11] structures. Olkhovsky, Recami, and Salesi came out with the idea that for *non-resonant* tunneling through two potential barriers, the tunneling time (which is a phase time) is independent not only of the barrier width but also of the barrier separation [8]. The approximations introduced in this reference to obtain the unknown coefficients, led these authors to unphysical results like the generalized Hartman effect. This has been called the generalized Hartman effect (GHE). The two-barrier problem can be solved without approximations, see for example, in the work of Pereyra [12]. An experiment to check this effect was performed by Longhi et al. [10] where optical pulses of 1,550 nm wavelength were transmitted through a double-barrier system of Bragg gratings. In this reference, non-conclusive and inappropriately presented results for five different values of the gratings separation were reported. Most of the theoretical conclusions were based on questionable formulas and unnecessarily involved calculations. For example, Equation 2 (used in Equations 3 and 4) of [8] is not the actual transmission coefficient through a double Bragg grating. A criticism on the mathematical rigor on GHE is also given by S. Kudaka and S. Matsumoto [13,14]. It is easy to check from a straightforward calculation, or from the precise and general formulas published in [7] as quoted below, that the phase time for a double barrier (DB) with separation *L* has the structure

(1) $$\begin{array}{lll} \tau \; & =\;T_{2}\frac{2L}{T}\frac{\text{dk}}{\mathrm{d\omega}}+T_{2}A_{i}(F\text{sin}\text{kL}+G\text{cos}\text{kL})\; & \;\\ & \;\;+T_{2}A_{r}(F\text{cos}\text{kL}+G\text{sin}\text{kL})\; \end{array}$$

with *T*_2_ and *T* the double- and single-barrier transmission coefficients, respectively, *k* the wave number, *ω* the frequency and *A*_*i*_, *A*_*r*_, *F*, and *G* simple functions of the potential parameters (P. Pereyra and H. P. Simanjuntak, unpublished work). Despite this clear dependence on *L*, involved and contradictory arguments lead to establish that *τ* becomes independent of *L*[8,10,11]. In the following we will consistently use *a* for the separation between barriers.

For the inference of a generalized Hartman effect to be meaningful for multi-barriers, double superlattices (SLs) or double Bragg gratings (BG), one would of course need to keep the physical parameters [like the energy (wavelength) of the particle (wave)] fixed as the length between barriers is increased. The tunneling and transmission times behavior should be taken with care when one tries to find a Hartman effect due to barrier separation in multi-barrier systems [8,11] since, in general, the density of resonance energies grows rapidly as the separation increases. It is well known that the non-resonant gaps in the band structure of a SL or a BG become resonating when these systems are divided and separated; and the separation is increasingly varied. This was already recognized in [15] (for double SL) and in [10] (for double BG). On the other hand, it is well known that the tunneling time follows the resonant band structure [7,16]. Thus, it is not possible to keep increasing the separation between barriers and superlattices without crossing resonances. For this reason, visualized here with specific examples for electrons and electromagnetic waves, the existence of a generalized Hartman effect is a rather questionable issue. For these examples we perform first principle calculations using the *actual* transmission coefficient of the system (such as that of double BG in the experiment in [10]) so that we can justify completely that the so-called generalized Hartman effect is erroneous.

To study the Hartman effect and to criticize the presumption of a generalized Hartman effect in superlattices, Bragg gratings, and multi-barrier systems, we will use the theory of finite periodic system that allows straightforward calculation of the phase time. For electron tunneling, we shall assume periodic and sectionally constant potentials with cells of length *ℓ*_*c*_=*a*+*b* and a barrier of width *b* and strength *V*_*o*_ in the middle. For electromagnetic waves, each cell consisting of dielectrics 1 and 2 will contain a dielectric 2 of length *b* in the middle. In this case *ϵ*_*i*_, *n*_*i*_, and *μ*_*i*_ (with *i*=1,2) are the corresponding permittivities, refractive indices, and permeabilities; the regions outside the SL are assumed to be air. For Bragg gratings, the refractive indices are periodic.

## Methods

If we have a Gaussian wave packet (of electrons or electromagnetic waves) through a SL of length *nℓ*_*c*_−*a*, the centroid phase time (which is taken here as the tunneling or transmission time) is given by [7,17,18]

(2) $$\begin{array}{lll} \tau_{n}(E)\; & =\;-\mathrm{a\hbar}\frac{\text{dk}}{\text{dE}}+\frac{\hbar }{|\alpha_{n}{|}^{2}}\left\{\frac{1}{2}U_{2n-1}(\alpha_{R})\frac{d\alpha_{I}}{\text{dE}}\right)\; & \;\\ & \;\;\left(-\frac{\alpha_{I}}{\left(1-\alpha_{R}^{2}\right)}\left[n-\frac{\alpha_{R}}{2}U_{2n-1}(\alpha_{R})\right]\frac{d\alpha_{R}}{\text{dE}}\right\}.\; \end{array}$$

Here *α*=*α*_*R*_+*iα*_*I*_ is the (1,1) element of the single-cell transfer matrix *M*; *U*_*n*_(*α*_*R*_) are the Chebyshev polynomials of the second kind evaluated at *α*_*R*_; and *α*_*n*_ is the (1,1) element of the *n*-cell transfer matrix *M*_*n*_. This is given by [16]

(3) $$\alpha_{n}\;=\;U_{n}(\alpha_{R})-\alpha^{*}U_{n-1}(\alpha_{R}).$$

At *resonance*, where *U*_*n*−1_=*U*_2*n*−1_=0, we have [16]

(4) $$\alpha_{R}=\text{cos}\left(\frac{\nu \Pi }{n}\right)\;\nu =0,\pm 1,\ldots \;$$

The expression for the tunneling or transmission time simplifies as

(5) $$\tau_{n,\text{res}}\;=\;-a\sqrt{\frac{m_{A}}{2E}}-\frac{\mathrm{n\hbar}\alpha_{I}}{\left(1-\alpha_{R}^{2}\right)}\frac{d\alpha_{R}}{\text{dE}}.$$

The tunneling time in Equation 2 is exact and general and valid for arbitrary number of cells, barrier width, and barrier separation. Thus, one can check the existence or not of a (generalized) Hartman effect at will. For concrete examples, we consider superlattices like (GaAs/Al_0.3_Ga_0.7_As)^*n*^/GaAs, with electron effective mass *m*_A_=0.067 *m* in GaAs layers, *m*_B_=0.1 *m* in Al_0.3_Ga_0.7_As layers (*m* is the bare electron mass) and *V*_o_=0.23 eV, and Bragg gratings with periodic refractive index.

## Results and discussion

### Electron tunneling

If we consider electrons through superlattices with unit cell length *ℓ*_*c*_=*a*+*b*, we will have

(6) $$\alpha \equiv M_{11}=\text{exp}[\text{ika}](\cosh\text{qb}-i(q^{2}-k^{2})\sinh\text{qb}/2\text{qk})$$

with $k=\sqrt{2m_{A}E}/\hbar$ and $q=\sqrt{2m_{B}(V_{\text{o}}-E)}/\hbar$. When *m*_*A*_, *m*_*B*_ and *V*_o_ are taken as fixed parameters, we choose *a*=100 Å and *b*=30 Å.

**Figure 1:**
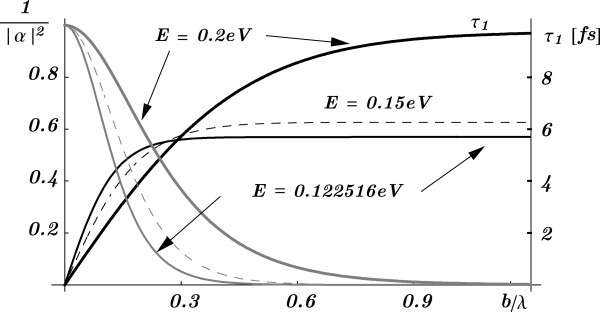
**The single-barrier transmission coefficient and the tunneling time.** The single-barrier transmission coefficient 1/|*α*|^2^ (gray lines) and the tunneling time *τ*_1_ (dark lines) as functions of the reduced barrier width *b*/*λ*, when the electron energies are *E*=0.122516 eV, *E*=0.15 eV and *E*=0.2 eV. In the tunneling time curves, the Hartman effect is evident.

For a *single* barrier, *n*=1, the tunneling time *τ*_1_ plotted in Figure 1 as a function of the reduced barrier width *b*/*λ* shows the well-known Hartman effect. The energy *E* is kept fixed and $\lambda =2\Pi \hbar /\sqrt{2m_{A}E}$ is the de Broglie wavelength.

With *α*_*R*_ and *α*_*I*_ growing exponentially with the barrier width *b*, one can easily show from Equation 2 that for large *b*, the non-resonant tunneling time approaches that for a single barrier, i.e., *τ*_*n*_(*E*)≈*τ*_1_(*E*) as

(7) $$\tau_{n}(E)\approx \frac{\hbar }{\sqrt{(V_{o}-E)E}}\frac{V_{o}\sqrt{m_{A}/m_{B}}}{V_{o}-E(1-m_{A}/m_{B})}.$$

This is the well-known Hartman effect. Since this quantity becomes also independent of the barrier separation [8,11]*a*, it has been taken as the analytical evidence of a generalized Hartman effect. However, such an approximation that leads to the independence on *a* and *n* is obtained by taking the limit of large *b* first that is strictly speaking infinite, which makes the first barrier the only one that matters for the incoming wave to penetrate while the rest of the SL is immaterial. This was also pointed out by Winful [9]. However, Winful [9] used an approximation: The transmission of the double square barrier potential to model the transmission through the double BG. Here, we present calculations using the *actual* transmission coefficient through the double BG. As mentioned before, for the generalized Hartman effect to be meaningful, it should not matter whatever limit we take first whether on *a*, *b*, or *n*. It turns out that a non-resonant energy region becomes resonant as the separation *a* increases (see the discussion on the double Bragg gratings in section ‘Hartman effect in two Bragg gratings systems’).

**Figure 2:**
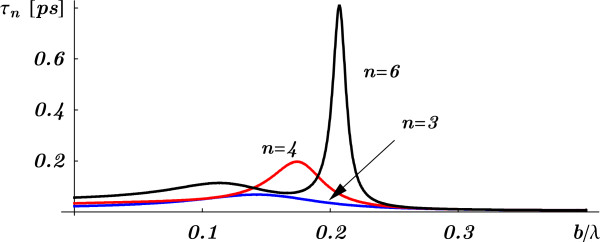
**The tunneling time *τ***_***n***_** as a function of the reduced barrier width.** The tunneling time *τ*_*n*_ as a function of the reduced barrier width *b*/*λ* for electrons (with energy *E*=0.15 eV) through superlattices with *n*=3,4, and 6.

The situation is completely different for *resonant* tunneling through a SL with *large but finite* barrier width *b* where Equation 5 shows that the tunneling time becomes *τ*_*n*_(*E*)∝*be*^2*qb*^ (since *α*_*R*_ and *α*_*I*_ behave as *e*^*qb*^ for large *b*). Thus, relatively small barrier width would be needed to study the effect of the barrier separation and the number of barriers on the tunneling time. The tunneling time for a relatively small barrier width is shown in Figure 2 for an electron (with energy *E*=0.15 eV) through SLs which number of cells are *n*=3,4, and 6.

**Figure 3:**
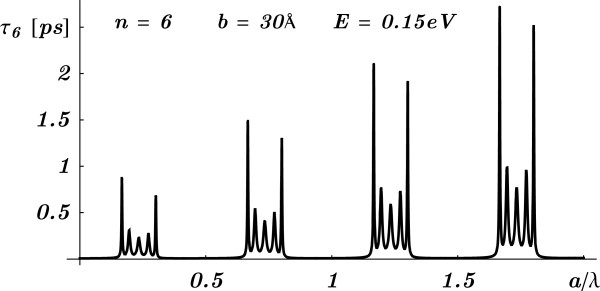
**The tunneling time *τ***_***6***_** as a function of reduced barrier separation and fixed barrier width.** The tunneling time *τ*_6_ as a function of reduced barrier separation *a*/*λ* for fixed barrier width *b*, number of cells *n*=6 and electron energy *E*=0.15 eV with the corresponding de Broglie wavelength *λ*.

Looking at *α*_*R*_ and *α*_*I*_, that are oscillating functions in *a*, it is clear that it is *not* possible to have the tunneling time to be independent of the barrier separation *a*, by keeping the barrier width and number of cells fixed. Therefore, the so-called generalized Hartman effect is at least dubious. The tunneling time behavior that will be found below for the double BG is easy to understand here. Starting with a certain barrier separation *a*, a non-resonant phase time becomes a resonant one as *a* is increased, while the other parameters are kept fixed. This is shown in Figure 3 where the tunneling time is plotted as a function of the reduced barrier separation, *a*/*λ*, for fixed *b*, *n*, and electron energy *E*. This result shows that in this kind of systems, the presumption of a generalized Hartman effect is incorrect.

**Figure 4:**
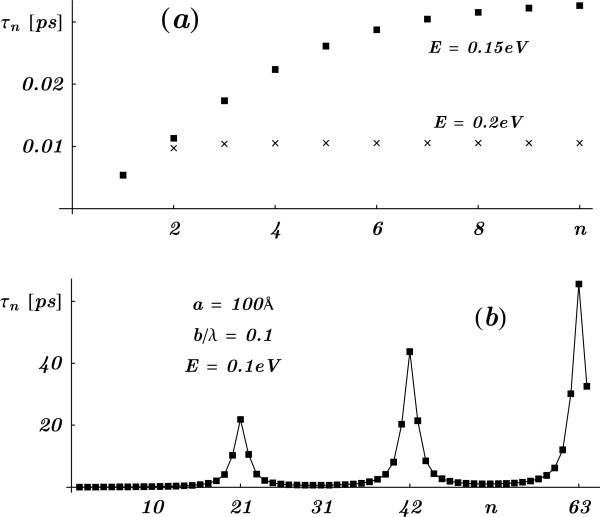
**The tunneling time *τ***_***n***_** as the number of cells*n* in a SL is varied.** (**a**) Saturation of *τ*_*n*_ for electron energies *E*=0.15 eV and *E*=0.2 eV in the gap. (**b**) The energy is close to a resonant band-edge. In this case, more resonances appear as *n* is increased with the energy fixed. No Hartman effect can be inferred from this figure.

The Hartman effect as a consequence of varying the number of cells was already discussed in [7]. In Figure 4 we show three qualitatively different examples on the behavior of the tunneling time as a function of *n*. In Figure 4a for energies in the gap (*E*=0.15 eV and *E*=0.2 eV), the saturation of the tunneling time exhibits the well-known Hartman effect. In Figure 4b, the energy lies at the edge of a resonant region. The phase time *τ*_*n*_ resonates for multiples of *n*=21. This behavior is clearly understood if we consider Equations 4 and 5. Equation 4 implies that the *same* resonance energy $E_{\nu }^{n}$ is found for different number of cells as long as the ratio *ν*/*n* is constant. This means that $E_{1}^{n}=E_{2}^{2n}=E_{3}^{3n}=\ldots$. From Equation 5, it is also evident the linear dependence of *τ*_*n*_ on *n*.

### The Hartman effect and the electromagnetic waves

Electromagnetic waves have been used for discussions on the Hartman effect [9]. For a superlattice *L*(*H*/*L*)^*n*^ made of alternating layers with refractive indices *n*_*L*_ and *n*_*H*_, the phase time (PT) for each frequency component of a Gaussian wave packet through a SL of length *nℓ*_*c*_−*a* is also obtained from Equation 2 with *k*_*L*,*H*_=*ωn*_*L*,*H*_/*c* and with [7]

(8) $$\alpha_{R}=\text{cos}k_{L}a\text{cos}k_{H}b-\frac{n_{L}^{2}+n_{H}^{2}}{2n_{H}n_{L}}\text{sin}k_{L}a\text{sin}k_{H}b,$$

(9) $$\alpha_{I}=\text{cos}k_{L}a\text{sin}k_{H}b+\frac{n_{L}^{2}+n_{H}^{2}}{2n_{H}n_{L}}\text{sin}k_{L}a\text{cos}k_{H}\mathrm{b.}$$

**Figure 5:**
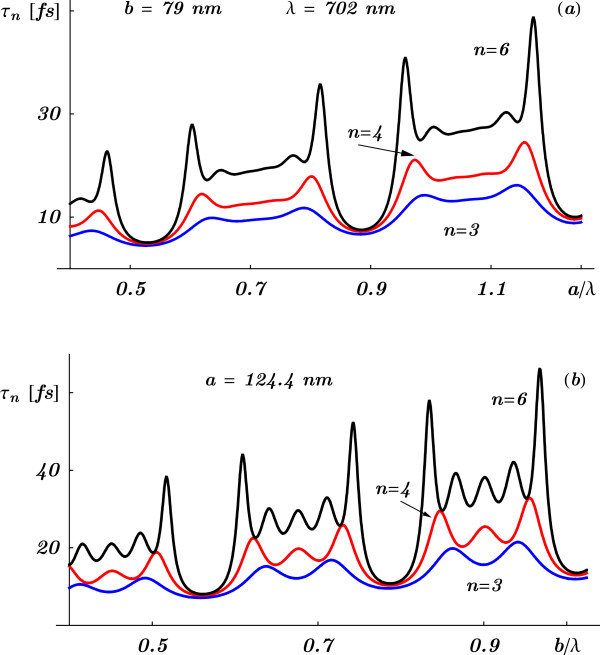
**The phase times *τ***_***n***_** as functions of the reduced thicknesses.** The phase times *τ*_*n*_ as functions of the reduced thicknesses *a*/*λ* (**a**) and *b*/*λ* (**b**) for a superlattice (*L*/*H*/*L*)^*n*^ made of fused silica (L) (*n*_*L*_=1.41) and titanium oxide (H) (*n*_*H*_=2.22) for *λ*=702 nm.The oscillating behavior of *τ*_*n*_ with clear band structure is far away of any Hartman effect.

To see the effect of varying the size of the SL on the PT, one has to be sure that such variation will still keep the wavelength inside a photonic band gap. It was shown that by increasing the number of cells, for fixed thicknesses of layers and wavelength in a gap, the PT exhibits [7] the observed Hartman effect [2,3]. However, this condition will not be possible by varying arbitrarily the thicknesses of the layers. The reason is that there is only a small range of thicknesses that one can use to keep the chosen wavelength to lie in a gap before going out of it and may even reach resonances, as shown in Figure 5 where the PT oscillates (with a band structure) and grows as a function of the reduced thicknesses *a*/*λ* and *b*/*λ*. This is analogous to the electron tunneling time shown in Figure 3.

#### Hartman effect in two Bragg gratings systems

**Figure 6:**
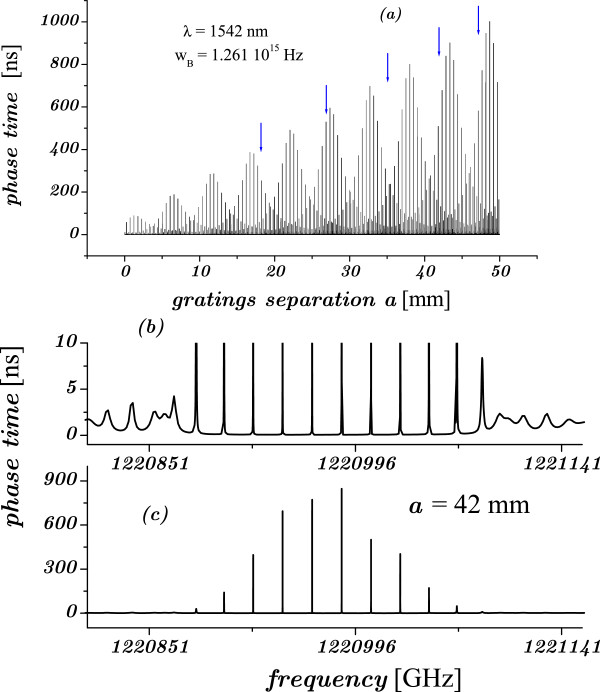
**The phase time as a function of the Bragg gratings separation.** (**a**) The phase time as a function of the separation *a* between two Bragg gratings, for incident *λ*=1,542 nm, *k*_B_=6.1074/*μ*m, *n*_0_=1.452, *n*_1_/*n*_0_=1.8×10^−4^, and *L*_o_=8.5 mm. (**b, c**) The PT is plotted as a function of *ω*, for *a*=42 mm. The phase time in (b) is the same as that in (c) but plotted from 0 to 10 ns to compare with Figure 2 in [10]. Arrows indicate the *a*s in [10].

We now consider the system that was taken in [10] as thought to support the idea of a generalized Hartman effect: the double Bragg gratings (DBG). Independent of the approximate method used in that paper, we find that assuming sin(*k*_B_*a*)=0 (the only way to obtain the reduced expressions of Table 1 in [10]) and still considering *a* as a variable are incongruous. Moreover, the idea that the PT becomes independent of *a* is incompatible with the Equation (4b) in their work, where a linear dependence on *a* is reported. In the DBG, the gratings of length *L*_*o*_ and refractive index *n*(*z*)=*n*_0_+*n*_1_ cos(2*k*_B_*z*) are separated by a distance *a*. The values of *a* considered in the experiment are indicated by arrows in Figure 6. The BG wave equation

(10) $$\frac{d^{2}\psi_{i}(z)}{dz^{2}}+k^{2}n^{2}(z)\psi_{i}(z)=0,$$

when ignoring the (*n*_1_/*n*_0_)^2^ term for *n*_1_/*n*_0_≪1 (as in [10]), becomes the Mathieu equation, in which solutions *ψ*_1_(*z*)=Se(*u*,*v*;*k*_B_*z*+*Π*/2) and *ψ*_2_(*z*)=So(*u*,*v*;*k*_B_*z*+*Π*/2) are Mathieu functions [19] with $u=\left(1+2n_{1}/n_{0}\right)n_{0}^{2}k^{2}/k_{\text{B}}^{2}$ and $v=2\sqrt{n_{0}n_{1}}k/k_{\text{B}}$. The real and imaginary parts of the (1,1) element of the transfer matrix are

(11) $$\begin{array}{lll} \alpha_{R}\; & =\;A\frac{\text{cos}kn_{0}a}{W^{2}}+B\frac{\text{sin}kn_{0}a}{W^{2}}\; & \;\\ \alpha_{I}\; & =\;C\frac{\text{cos}kn_{0}a}{W^{2}}+D\frac{\text{sin}kn_{0}a}{W^{2}}\; \end{array}$$

with *W* the Wronskian and

(12) $$\begin{array}{lll} A\; & =\;\theta_{2}\theta_{1}+\mu_{2}\nu_{1}+\nu_{2}\mu_{1}+\chi_{2}\chi_{1}\; & \;\\ B\; & =\;\frac{1}{kn_{0}}\left(\theta_{2}\nu_{1}+\nu_{2}\chi_{1}\right)-kn_{0}(\mu_{2}\theta_{1}+\chi_{2}\mu_{1})\; & \;\\ C\; & =\;kn_{0}(\theta_{2}\mu_{1}+\mu_{2}\chi_{1})-\frac{1}{kn_{0}}\left(\nu_{2}\theta_{1}+\chi_{2}\nu_{1}\right)\; & \;\\ D\; & =\;\theta_{2}\chi_{1}+\chi_{2}\theta_{1}-k^{2}n_{0}^{2}\;\;\mu_{2}\mu_{1}-\frac{1}{k^{2}n_{0}^{2}}\;\;\nu_{2}\nu_{1}.\; \end{array}$$

Here *θ*_1_=*θ*(*L*_*o*_,0), *θ*_2_=*θ*(2*L*_*o*_+*a*,*L*_*o*_+*a*) analogously for *χ*_1,2_, *μ*_1,2_, *ν*_1,2_, with

(13) $$\begin{array}{lll} \theta (z_{2},z_{1})\; & =\;\psi_{1}(z_{2})\frac{d\psi_{2}(z_{1})}{\text{dz}}-\psi_{2}(z_{2})\frac{d\psi_{1}(z_{1})}{\text{dz}}\; & \;\\ \chi (z_{2},z_{1})\; & =\;\psi_{1}(z_{1})\frac{d\psi_{2}(z_{2})}{\text{dz}}-\psi_{2}(z_{1})\frac{d\psi_{1}(z_{2})}{\text{dz}}\; & \;\\ \mu (z_{2},z_{1})\; & =\;\psi_{1}(z_{1})\psi_{2}(z_{2})-\psi_{2}(z_{1})\psi_{1}(z_{2})\; & \;\\ \nu (z_{2},z_{1})\; & =\;\frac{d\psi_{1}(z_{2})}{\text{dz}}\frac{d\psi_{2}(z_{1})}{\text{dz}}-\frac{d\psi_{2}(z_{2})}{\text{dz}}\frac{d\psi_{1}(z_{1})}{\text{dz}}.\; \end{array}$$

Using parameters of Longhi et al. [10] for *n*_0_,*n*_1_, *k*_B_, and *L*_*o*_, the non-resonant gap becomes resonant as the gratings separation increases. Though details are beyond the purpose of this paper, we plot in Figure 6 the PT as a function of the separation *a* for incident-field wavelength *λ*=1542 nm, and as a function of the frequency *ω*, for *a*=42 mm. Recall that in [10], *λ*≃1,550 nm was considered. While the PT appears completely in graph (c), in (b) it is plotted in a different range to compare with the experiment. The resonant behavior of the PT with *a* and the absence of any generalized Hartman effect are evident. Similar results are obtained when *λ*=2*Π*/*k*_*B*_.

## Conclusion

We have shown that the presumption of generalized Hartman effect for tunneling of particles and transmission of electromagnetic waves is not correct.

## Competing interests

The authors declare that they have no competing interest.

## Authors’ contributions

HPS initialized the work. Both authors carried out the calculations. Both authors read and approved the final manuscript.
